# In vivo evidence that truncated trkB.T1 participates in nociception

**DOI:** 10.1186/1744-8069-5-61

**Published:** 2009-10-29

**Authors:** Cynthia L Renn, Carmen C Leitch, Susan G Dorsey

**Affiliations:** 1 School of Nursing, University of Maryland, Baltimore, MD 21201, USA; 2 Program in Neuroscience, University of Maryland, Baltimore, MD 21201, USA; 3 Program in Oncology, University of Maryland, Baltimore, MD 21201, USA

## Abstract

Brain-Derived Neurotrophic Factor (BDNF) is a central nervous system modulator of nociception. In animal models of chronic pain, BDNF exerts its effects on nociceptive processing by binding to the full-length receptor tropomyosin-related kinase B (trkB.FL) and transducing intracellular signaling to produce nocifensive behaviors. In addition to trkB.FL, the trkB locus also produces a widely-expressed alternatively-spliced truncated isoform, trkB.T1. TrkB.T1 binds BDNF with high affinity; however the unique 11 amino acid intracellular cytoplasmic tail lacks the kinase domain of trkB.FL. Recently, trkB.T1 was shown to be specifically up-regulated in a model of HIV-associated neuropathic pain, potentially implicating trkB.T1 as a modulator of nociception. Here, we report that trkB.T1 mRNA and protein is up-regulated in the spinal dorsal horn at times following antiretroviral drug treatment and hind paw inflammation in which nocifensive behaviors develop. While genetic depletion of trkB.T1 did not affect baseline mechanical and thermal thresholds, the absence of trkB.T1 resulted in significant attenuation of inflammation- and antiretroviral-induced nocifensive behaviors. Our results suggest that trkB.T1 up-regulation following antiretroviral treatment and tissue inflammation participates in the development and maintenance of nocifensive behavior and may represent a novel therapeutic target for pain treatment.

## Findings

BDNF is a potent modulator of pain processing in the CNS [[1,2]; for review see [3,4]]. BDNF exerts effects on nociception by binding to trkB.FL and initiating intracellular signaling cascades that lead to transcriptional changes. Noxious stimulation increases BDNF and trkB.FL production in the spinal dorsal horn [5-9] and brainstem [10,11], leading to hyperalgesia and the formation of nocifensive behaviors. Interfering with trkB.FL signaling via trkB.FL-specific exogenous antibody administration [12] or by blocking receptor activation in the chemical-genetic transgenic *trkB*^F616A ^mice [13] prevents the development of tissue- and nerve injury-induced thermal and mechanical hypersensitivity [14].

In addition to trkB.FL, the *Ntrk2 *(trkB) locus encodes for several alternatively-spliced isoforms of the receptor [15], including trkB.T1, the predominant isoform expressed in the adult mammalian nervous system. The extracellular domain of trkB.T1 is identical to the full-length isoform, which enables high-affinity BDNF binding [15,16]. However, the 11 amino acid intracellular portion of trkB.T1 lacks the kinase activation domain necessary to activate classical signal transduction pathways. Since trkB.T1 heterodimerization with trkB.FL inhibits trans-autophosphorylation of the trkB.FL kinase domain, studies support a model in which trkB.T1 functions to reduce BDNF signaling [17-22]. However, we [23,24] and others [25-31] have demonstrated evidence that trkB.T1 may signal independently.

TrkB.T1 is up-regulated in pathological states including human Alzheimer's disease [32] and it is the mechanism underlying premature hippocampal cell death in a mouse model of Down Syndrome [23,33]. Moreover, altered trkB.T1 expression can affect synaptic plasticity. Over-expression of trkB.T1 in hippocampal neurons leads to the inhibition of synaptic potentiation [34] and over-expressing trkB.T1 at neuromuscular synapses results in disassembly of acetylcholine receptor clustering [18]. Recently, trkB.T1 specifically was shown to be significantly up-regulated in dorsal root ganglion neurons in a rodent model of HIV-associated neuropathic pain [35]. Thus, it is reasonable to hypothesize that trkB.T1 up-regulation might have a specific role in pain processing.

**Figure 1:**
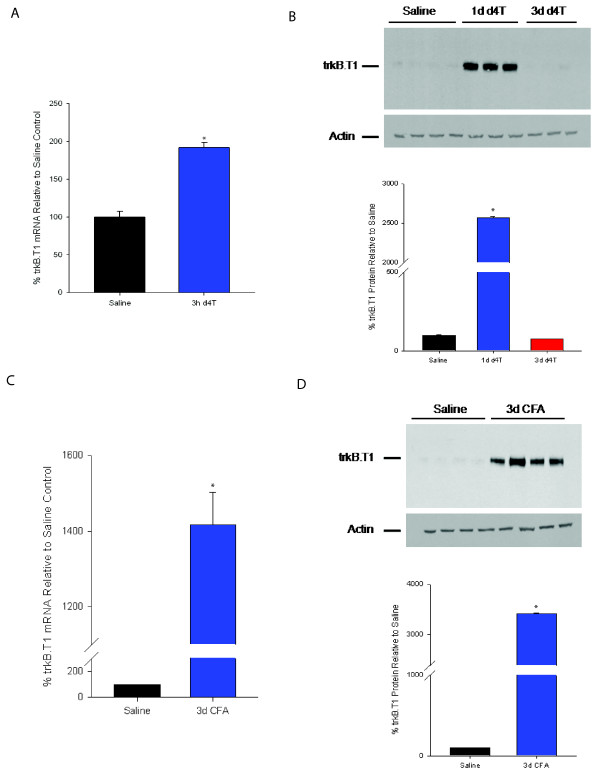
**TrkB.T1 expression in the spinal dorsal horn of d4T and CFA-treated mice A**. Quantification of trkB.T1 mRNA expression in the dorsal horn of wildtype mice 3 hours after receiving a saline tail injection (black bar; n = 6) or an injection of d4T [50 mg/kg] (blue bar; n = 6). **B**. TrkB.T1 protein expression in the dorsal horn in the antiretroviral model was assayed via western blot. The top panel shows a representative western blot of trkB.T1 following saline or d4T injection at 1d and 3d. Below the panel the results from saline treated (black bar; n = 6) and 1d (blue bar; n = 6) or 3d (red bar; n = 6) after d4T treatment are quantified. *indicates p < 0.05 by ANOVA with Tukey post-hoc testing. **C**. Quantification of trkB.T1 mRNA expression in the dorsal horn of wildtype mice 3 days after receiving a saline hind paw injection (black bar; n = 6) or an injection of CFA (blue bar; n = 6). **D**. TrkB.T1 protein expression in the dorsal horn in the CFA model was assayed via western blot. The top panel shows a representative western blot of trkB.T1 following a saline or CFA hind paw injection at 3d. Below the panel the results from saline treated (black bar; n = 6) and 3d CFA (blue bar; n = 6) are quantified. *indicates p < 0.05 by t-test.

First, we examined trkB.T1 mRNA expression in the spinal dorsal horn of a mouse model of antiretroviral-associated neuropathic pain [36], a model in which significant mechanical hypersensitivity occurs one day after tail vein injection with stavudine (d4T) [37]. Since behavioral changes occur soon after treatment [37], we examined early time points and found that trkB.T1 mRNA was significantly up-regulated at 3 hours after tail vein injection of d4T compared with mice injected with an equivalent volume of saline vehicle (Figure 1A). At later time points (6 h, 12 h, 18 h, 3 d) trkB.T1 mRNA levels returned to saline control levels (data not shown). Next, we asked whether trkB.T1 protein was regulated in the dorsal horn after treatment. In Figure 1B, we demonstrate that trkB.T1 protein is significantly up-regulated one day after d4T injection compared with saline control-injected animals. By three days after d4T treatment, trkB.T1 levels returned to saline control levels, a result consistent with our mRNA data. We next asked whether trkB.T1 was up-regulated in the dorsal horn of mice with hind paw inflammation. As demonstrated in Figures 1C and 1D, both trkB.T1 mRNA and protein are significantly up-regulated in CFA-treated mice compared with mice receiving a saline control-injected hind paw. These results raise the possibility that the trkB.T1 receptor is involved in nociception in these two pain models.

**Figure 2:**
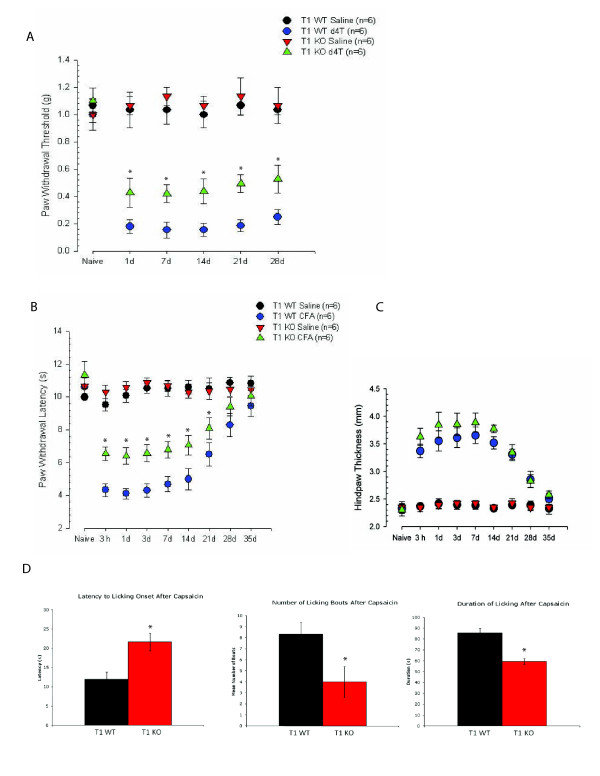
**Nocifensive behavior in trkB.T1 wildtype and knockout mice**. **A**. Mice were baseline tested for mechanical threshold and then randomized within genotype (wildtype, knockout; n = 12 per genotype) to receive a saline (n = 6 per genotype) or d4T (n = 6 per genotype) tail vein injection. Following tail vein injection, mice were tested at 1, 7, 14, 21 and 28d for the presence of mechanical sensitivity. * indicates T1 WT (blue circles) and T1 KO (green triangles) significantly different from saline controls; p < 0.05 by Repeated Measures ANOVA with Tukey post-hoc testing. **B**. Mice were baseline tested for thermal threshold and then randomized within genotype (wildtype, knockout; n = 12 per genotype); to receive saline (n = 6 per genotype) or CFA (n = 6 per genotype) hind paw injection. Following hind paw injection, mice were testing for thermal sensitivity at 3 h and 1, 3, 7, 14, 21, 28 and 35d.* indicates T1 WT (blue circles) and T1 KO (green triangles) significantly different than saline controls; p < 0.05 by Repeated Measures ANOVA with Tukey post-hoc testing. **C**. The hind paws from mice in each group tested in B. were measured using calipers and the thickness recorded in mm. **D**. Latency to licking, number of licking bouts and duration of licking following capsaicin administration were recorded and the mean and s.e.m. presented for each genotype (n = 6). *indicates p < 0.05 by t-test.

If trkB.T1 is up-regulated in the dorsal horn following noxious stimulation, then genetic depletion of trkB.T1 [23] would be expected to mitigate nocifensive behaviors in these models. We first tested this hypothesis in the antiretroviral model (i.e. stavudine) of mechanical hypersensitivity [37]. There were no differences in mechanical threshold (g) in naïve trkB.T1 null mice at baseline (Figure 2A). Genetic deletion of trkB.T1 provided significant attenuation of mechanical hypersensitivity throughout the testing period (Figure 2A). Similarly, in the well-established CFA model of inflammation although we found no difference in thermal paw withdrawal latency (s) in trkB.T1 KO mice at baseline, the absence of trkB.T1 in vivo significantly attenuated thermal hypersensitivity (Figure 2B). In Figure 2C, we show that there were no differences in the degree of hind paw thickness secondary to CFA injection. As this is an indirect measure of inflammation we conclude that there was no difference in the inflammatory response in the trkB.T1 knockout mice. Next, we asked whether genetic deletion of trkB.T1 would provide a benefit for mice treated with capsaicin, an agonist of TRPV1 receptors that are expressed in BDNF-immunoreactive nociceptors. Similar to results in our previous two models, the absence of trkB.T1 provided significant attenuation of the latency to lick, number of licking bouts and duration of licking after capsaicin treatment. Thus, we conclude that the absence of trkB.T1 provides significant protection against the development of thermal and mechanical hypersensitivity.

BDNF signaling modulates nociceptive processing across a variety of pre-clinical models of pain (for review, [38]). Most studies have focused on trkB.FL as the receptor responsible for BDNF-mediated nociception, since antibodies specific for trkB.FL [12] and abrogation of trkB.FL activation using the *trkB*^F616A ^transgenic mouse [14] attenuate thermal and mechanical hypersensitivity. Few studies, however, have examined a specific role for trkB.T1. Yajima et al. [12] concluded that trkB.T1 did not have a role in nociception since repeated intrathecal injections with anti-trkB.T1 did not produce changes in nerve injury-induced thermal hyperalgesia. However, we (Dorsey and Renn, unpublished) and others (Dr. Rita Balice-Gordon, University of Pennsylvania, personal communication) have shown that the trkB.T1 antibody used in the Yajima et al. [12] study cross-reacts with other epitopes *in vivo*. Moreover, as acknowledged in Wang et al. [14], indirect methods of targeting receptor activation, for example via exogenous antibody administration, can be inconclusive. In support of a role for trkB.T1 in nociception, a recent microarray study in a model of HIV-associated neuropathic pain found that trkB.T1, but not trkB.FL or trkB.T2 (a second truncated isoform), was up-regulated in dorsal root ganglion neurons [35].

Since trkB.T1 and trkB.FL are presumably regulated by a common promoter, we also undertook a careful examination of spinal dorsal horn trkB.FL mRNA and protein regulation in the antiretroviral and inflammatory models in both wildtype and trkB.T1 knockout animals. As demonstrated in Additional File 1, we detected no significant regulation of trkB.FL mRNA (panels A-D) following antiretroviral or inflammatory treatment compared with vehicle controls in either genotype. Similar results were found for protein levels (panels E-H), although trkB.FL was down-regulated approximately 25% in wildtype CFA-treated animals. Thus, we conclude that the up-regulation of trkB.T1 following noxious stimulation is specific for this isoform.

Here, we demonstrate that trkB.T1 mRNA and protein are both significantly up-regulated in the spinal dorsal horn following systemic antiretroviral drug administration and hind paw inflammation (Figure 1). We show that *in vivo *deletion of trkB.T1 did not affect acute nociceptive processing, since baseline mechanical and thermal thresholds were not significantly different from wildtype strain-matched controls (Figure 2). However, following antiretroviral administration or hind paw inflammation via CFA injection or capsaicin treatment, animals lacking trkB.T1 were significantly protected against the development of mechanical and thermal hypersensitivity (Figure 2A, B, D). These results suggest that trkB.T1 participates in the development and maintenance of mechanical and thermal hypersensitivity, and that upregulation specifically contributes to the degree of thermal and mechanical hypersensitivity since the null mutant demonstrates attenuated hypersensitivity to our noxious stimulation paradigms.

The expression of trkB.FL and trkB.T1 is conserved across species and throughout evolution, and trkB isoform expression is regulated during development when the dominant trkB isoform expression in the brain switches from trkB.FL to trkB.T1 [39]. Receptor expression is cell specific and not all cells express both isoforms. For example, neurons express both isoforms [33] while astrocytes only express trkB.T1 [23,40]. The prevailing view has been that trkB.T1 expression leads to dominant negative inhibition of full-length trkB signaling and a reduction in the activation of downstream signaling molecules [17-23,33]. If trkB.T1 were functioning solely as a dominant negative inhibitor of signaling in the dorsal horn, the prediction would be that deletion of trkB.T1 would result in more, not less pain. However, in the animal models of pain tested in this study, genetic depletion of trkB.T1 results in attenuation of pain. Several studies have provided evidence to suggest that trkB.T1 can signal independently, however the precise nature and functional relevance of this signal has been less clear. The available evidence regarding trkB.T1 signaling suggests that BDNF binding to trkB.T1 causes increased intracellular calcium release [28]. We have shown that neurons from a mouse model of neurodegeneration over-express trkB.T1, resulting in a significant increase in resting intracellular calcium; reducing trkB.T1 expression in those neurons reduced intracellular calcium to wildtype levels [23]. In a rodent model of antiretroviral-induced neuropathy, intrathecal injection of TMB-8, a drug that buffers calcium, provided near complete abrogation of mechanical hypersensitivity [36]. Thus, our results demonstrating attenuation in thermal and mechanical sensitivity in the absence of trkB.T1 could be because increased trkB.T1 expression induces increased intracellular calcium signaling which promotes hypersensitivity.

Our results support the idea that trkB signaling is complex, and that trkB.T1 participates in the development and maintenance of persistent pain. Genetic deletion of trkB.T1 provides significant protection from the development of thermal and mechanical hypersensitivity following noxious stimulation. Thus, we conclude that while previous studies have demonstrated a specific role for BDNF-mediated trkB.FL signaling in nociception, our results support a role for trkB.T1 in nociception that has not been previously described.

## Materials and methods

### Western blots

Procedures have been described elsewhere [23,33]. Primary antibodies included anti-trkB.T1 (Santa Cruz TrkB (C-13) sc-119), anti-trkB out (generous gift from David Kaplan, Hospital for Sick Kids, Toronto, Canada) and anti-actin (Sigma A2547). Secondary antibodies conjugated to peroxidase included anti-mouse IgG and anti-rabbit IgG (Amersham Pharmacia Biotech).

### qPCR

Methods for RNA extraction and qPCR techniques have been previously described (Dorsey et al., 2009). Primers specific for trkB.T1 included forward: 5'-TGG TGA TGT TGC TCC TGC TCA AGT-3'; reverse: 5'-CCC ATC CAG TGG GAT CTT ATG AAA C-3'. Primers specific for trkB.FL included forward: 5'-ACT TTG GCA TCA CCA ACA GTC AGC-3'; reverse: 5'-AGG TTG TAG CAC TCG GCA AGG AAA-3'. The β-actin gene was used as the reference gene with specific primers: forward: 5'-TGT GGT GCC AGA TCT TCT CCA TGT-3'; reverse: 5'-TGT GGT GCC AGA TCT TCT CCA TCT-3' (Integrated DNA Technologies).

### Generation of trkB.T1 wildtype and knockout mice

Mice were generated as previously described [23]. Wildtype and knockout experimental animals were the progeny of heterozygous trkB.T1 matings from animals at N13 generation.

### Nocifensive behavior

All experiments were approved by the University of Maryland Baltimore Institutional Animal Care and Use Committee and were performed following the guidelines of the International Association for the Study of Pain. All experiments were performed with the tester blind to the genotype of the mice and the treatment that they received. All mice tested were adult male mice (2-6 months of age weighing approximately 30 g. Details regarding the production of the inflammatory model using Complete Freund's adjuvant (CFA) can be found in Renn et al. [11] and the antiretroviral model methods are detailed in Dorsey et al. [37]. For capsaicin experiments, capsaicin (Sigma, St. Louis, MO) was dissolved in 100% dimethylsulfoxide (DMSO) followed by dilution with 0.9% saline to a concentration of 0.08 μg/μl. Using a 50 μl Hamilton syringe with a 1/2" 30-gauge needle attached, each mouse was given a 20 μl subcutaneous injection of capsaicin (1.6 μg/20 μl) into the plantar surface of the left hind paw. The mice were placed in individual Plexiglas cubicles and allowed to acclimate for approximately one hour before the injection of capsaicin. Licking induced by the capsaicin injection was used to indicate a nocifensive response. Each mouse was examined for a period of 5 minutes following the capsaicin injection by a blinded observer. The time (in seconds) to onset of licking, total accumulated time (in seconds) spent licking and the number of bouts of licking the capsaicin-injected paw was measured. Thermal Hyperalgesia: The Paw Thermal Stimulator (PTS, UARDG, Department of Anesthesiology, University of California, San Diego; attn George Ozaki) was used to assess thermal sensitivity. Mice were placed in individual Plexiglas cubicles on a glass surface maintained at a constant temperature of 30°C ± 0.1°C, and allowed to acclimate for approximately one hour prior to testing. Paw withdrawal latencies were defined as the time in seconds required from the onset of the stimulus to the point of a brisk withdrawal of the hind paw from the stimulus. An automatic 20-second cut-off was used to avoid tissue injury from prolonged exposure to the thermal stimulus. Each hind paw was tested three times and the response latency was the average of the three tests. Approximately 10 minutes elapsed between repeat test exposures of each hind paw to avoid tissue injury from the repeated applications of the thermal stimulus. Mechanical Allodynia: The nocifensive behavior of paw withdrawal from a mechanical stimulus was used to assess the development of mechanical allodynia. The mice were placed in individual Plexiglas cubicles on a wire mesh platform, and allowed to acclimate for approximately one hour. A series of von Frey filaments (Touch Test Sensory Evaluator Kit, myNeurolab.com, St. Louis, MO), with bending forces that ranged from 0.04 g to 1.40 g, was used to deliver the mechanical stimuli. Each filament was applied to the left hind paw until the filament just bent and was held in place for 5 seconds or until the mouse withdrew his paw. Each filament was tested 5 times on the left hind paw, with a period of 3-5 minutes elapsing between trials. A positive response to the stimulus was defined as a brisk withdrawal, with or without shaking or licking, of the hind paw during or immediately upon removal of the filament application. Using a procedure modified after Ren [41], the mechanical stimuli were applied to the plantar surface of the left hind paw, starting with the 0.4 g filament. If the 0.4 g filament elicited 3 positive responses out of 5 trials, then the mouse was tested moving downward through the series to the 0.04 g filament and the number of withdrawals was recorded for each filament. If the 0.4 g filament did not elicit 3 positive responses, then the mouse was tested moving upward through the series to the 1.4 g filament and the number of withdrawals was recorded for each filament. Threshold was defined as the filament with the lowest bending force that elicited at least 3 positive responses out of 5 trials.

## Competing interests

The authors declare that they have no competing interests.

## Authors' contributions

CLR assisted with the design of experiments, conducted the behavioral experiments and assisted with data analysis; CCL conducted the qPCR and western blot experiments; SGD designed and supervised the experiments, analyzed the data and wrote the manuscript. All authors have read and approved the final manuscript.

## Supplementary Material

Additional file 1 **trkB.FL mRNA and protein expression in the antiretroviral and inflammatory models in trkB.T1 WT and KO animals**. **A and B**. Quantification of trkB.FL mRNA expression in the dorsal horn of trkB.T1 wildtype mice (A) and trkB.T1 knockout mice (B) after receiving a saline tail injection (black bar; n = 3) or 1d (blue bar; n = 3) or 3d (red bar; n = 3) after an injection of d4T [50 mg/kg]. **C and D**. Quantification of trkB.FL mRNA expression in the dorsal horn of trkB.T1 wildtype mice (C) and trkB.T1 knockout mice (D) after receiving a saline hind paw injection (black bar; n = 3) or a hind paw injection of CFA (blue bar; n = 3). **E and F**. TrkB.FL protein expression in the dorsal horn of trkB.T1 wildtype (E) and trkB.T1 knockout (F) mice in the antiretroviral model was assayed via western blot. The top panels shows a representative western blot of trkB.FL following saline or d4T injection at 1d and 3d. Blots were stripped and re-probed with anti-actin to control for protein loading (shown in lower panel). Below the panel the results from saline treated (black bar; n = 3) and 1d (blue bar; n = 3) or 3d (red bar; n = 3) after d4T treatment are quantified. **G and H**. E and F. TrkB.FL protein expression in the dorsal horn in trkB.T1 wildtype (G) and trkB.T1 knockout (H) mice in the CFA model was assayed via western blot. The top panel shows a representative western blot of trkB.FL following a saline or CFA hind paw injection at 3d. Blots were stripped and re-probed with anti-actin to control for protein loading (shown in lower panel). Below the panel the results from saline treated (black bar; n = 3) and 3d CFA (blue bar; n = 3) are quantified. Click here for file
