# Supplementary figures and images for: In vivo evidence that truncated trkB.T1 participates in nociception
Source: Mol Pain. 2009 Oct 29;5:61. doi: 10.1186/1744-8069-5-61 (PMC2777863; doi:10.1186/1744-8069-5-61)

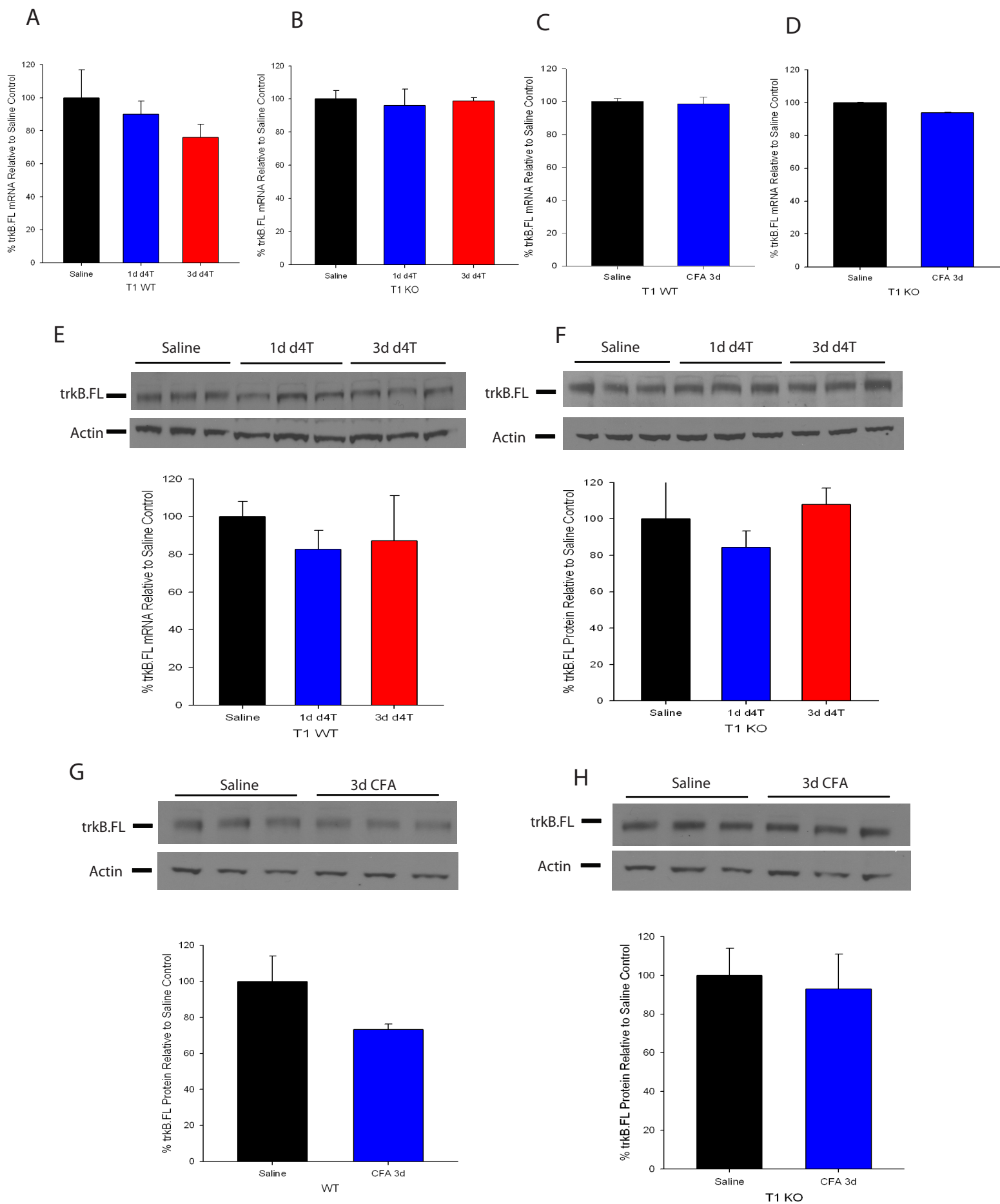

Supplement: Additional file 1 — trkB.FL mRNA and protein expression in the antiretroviral and inflammatory models in trkB.T1 WT and KO animals. A and B. Quantification of trkB.FL mRNA expression in the dorsal horn of trkB.T1 wildtype mice (A) and trkB.T1 knockout mice (B) after receiving a saline tail injection (black bar; n = 3) or 1d (blue bar; n = 3) or 3d (red bar; n = 3) after an injection of d4T [50 mg/kg]. C and D. Quantification of trkB.FL mRNA expression in the dorsal horn of trkB.T1 wildtype mice (C) and trkB.T1 knockout mice (D) after receiving a saline hind paw injection (black bar; n = 3) or a hind paw injection of CFA (blue bar; n = 3). E and F. TrkB.FL protein expression in the dorsal horn of trkB.T1 wildtype (E) and trkB.T1 knockout (F) mice in the antiretroviral model was assayed via western blot. The top panels shows a representative western blot of trkB.FL following saline or d4T injection at 1d and 3d. Blots were stripped and re-probed with anti-actin to control for protein loading (shown in lower panel). Below the panel the results from saline treated (black bar; n = 3) and 1d (blue bar; n = 3) or 3d (red bar; n = 3) after d4T treatment are quantified. G and H. E and F. TrkB.FL protein expression in the dorsal horn in trkB.T1 wildtype (G) and trkB.T1 knockout (H) mice in the CFA model was assayed via western blot. The top panel shows a representative western blot of trkB.FL following a saline or CFA hind paw injection at 3d. Blots were stripped and re-probed with anti-actin to control for protein loading (shown in lower panel). Below the panel the results from saline treated (black bar; n = 3) and 3d CFA (blue bar; n = 3) are quantified. [file 1744-8069-5-61-S1.pdf]
